# Automatic Change Detection for Real-Time Monitoring of EEG Signals

**DOI:** 10.3389/fphys.2018.00325

**Published:** 2018-04-04

**Authors:** Zhen Gao, Guoliang Lu, Peng Yan, Chen Lyu, Xueyong Li, Wei Shang, Zhaohong Xie, Wanming Zhang

**Affiliations:** ^1^Key Laboratory of High-Efficiency and Clean Mechanical Manufacture of MOE, National Demonstration Center for Experimental Mechanical Engineering Education, School of Mechanical Engineering, Shandong University, Jinan, China; ^2^School of Information Science and Engineering, Shandong Normal University, Jinan, China; ^3^Institute of Neurology, Shandong University, Jinan, China; ^4^Department of Neurology, Second Hospital of Shandong University, Jinan, China; ^5^Medical Imaging Center, Second Hospital of Shandong University, Jinan, China

**Keywords:** electroencephalogram (EEG), automatic change detection, real-time monitoring, joint features, martingale test

## Abstract

In recent years, automatic change detection for real-time monitoring of electroencephalogram (EEG) signals has attracted widespread interest with a large number of clinical applications. However, it is still a challenging problem. This paper presents a novel framework for this task where joint time-domain features are firstly computed to extract temporal fluctuations of a given EEG data stream; and then, an auto-regressive (AR) linear model is adopted to model the data and temporal anomalies are subsequently calculated from that model to reflect the possibilities that a change occurs; a non-parametric statistical test based on Randomized Power Martingale (RPM) is last performed for making change decision from the resulting anomaly scores. We conducted experiments on the publicly-available Bern-Barcelona EEG database where promising results for terms of detection precision (96.97%), detection recall (97.66%) as well as computational efficiency have been achieved. Meanwhile, we also evaluated the proposed method for real detection of seizures occurrence for a monitoring epilepsy patient. The results of experiments by using both the testing database and real application demonstrated the effectiveness and feasibility of the method for the purpose of change detection in EEG signals. The proposed framework has two additional properties: (1) it uses a pre-defined AR model for modeling of the past observed data so that it can be operated in an unsupervised manner, and (2) it uses an adjustable threshold to achieve a scalable decision making so that a coarse-to-fine detection strategy can be developed for quick detection or further analysis purposes.

## 1. Introduction

Electroencephalogram (EEG) reflects the electrical activity of the brain, which has become an important tool to record and comprehend the complex activities of the brain (Li et al., 2016). Among various applications, real-time EEG monitoring is a useful technique to observe the state of brain function and capture the potential fluctuations of brain activities. Practical examples of this technique include, but not limited to,

- Online monitoring of epileptic seizures using statistics of EEG (Yuan et al., 2013; Gajic et al., 2015). The mathematical model is established via observed EEG to determine whether the present status is normal by an online way.
- Change detection of brain status/pattern for patients with brain injury (O'Neill et al., 2015; Amorim et al., 2016), in which the real-time monitoring offers a continuous record of any seizure activity that may have been unwitnessed.
- Detection of sleep-disordered breathing events (Devuyst et al., 2010; D'Rozario et al., 2015), where real-time monitoring plays an important role to record any occurrence of sleep-related breathing disorders.
- Brain-computer interface (BCI) (Wang et al., 2012; Abdulkader et al., 2015). Obviously, it allows for real-time communication between the brain and the computer.

In real-time monitoring of EEG signals, one major goal is to find change(s) between brain states where the EEG signal changes from a normal state to the abnormal/ictal, for example, seizure onset detection of the epilepsy can be regarded as detection of statistical changes via monitoring EEG signals (Gao and Hu, 2013; Yan et al., 2015). In spite of that monitoring of EEG has been an extensively studied area in the literature (Chen et al., 2010; Mullen et al., 2015), change decision making in EEG signals is still a problem of challenge where EEG recordings are usually checked by experienced neurologists in an off-line operation in most of clinical diagnoses. Checking EEG recordings is a time-consuming and dull task that is prone to lower the accuracy and effectiveness of detection considering the massive amounts of collected data (Mporas et al., 2015), and meanwhile it brings a large delay because the manual observation can not achieve the real-time in monitoring. Moreover, results of detection would be different between neurologists because the determination largely depends on their subjective judgments/decisions (Boashash et al., 2015).

To address the drawbacks of manual decision making, plentiful approaches have been proposed over the decades to automatically detect the changes in monitoring EEG signals, and they can be divided into two groups. In the former group, machine learning is introduced into change detection of EEG signals (Liu et al., 2012; Wang et al., 2016). For example, Cloostermans et al. (2011) present a novel computer assisted EEG interpretation system that combines eight quantitative features into a single classifier and utilizes decision tree to obtain a classification per brain region, which may improve early detection of seizure activity and ischemia in critically ill patients. Tzallas et al. (2009) make good use of time-frequency analysis to represent the characteristics of different EEG segments and employ artificial neural networks to classify EEG segments for epileptic seizures. Moreover, Saghafi et al. (2017) employ cross channels maximum and minimum to monitor the EEG signals, then Multivariate Empirical Mode Decomposition and classification techniques are utilized to detect a possible change in the eye state. Although these approaches are experimentally fast and accurate when used for detecting possible changes, they need a supervised learning/training phase or prior knowledge to usage.

On the other hand, the latter group of statistical analysis based methods have been proposed where neither supervised learning/training nor prior knowledge can be applicable during EEG monitoring (Gao et al., 2010; Pachori and Bajaj, 2011). For example, Saaid et al. (2011) propose a change point detection for EEG signal application based on Particle Swarm Optimization (PSO). According to Kortelainen et al. (2012), a multiple change detection algorithm based on Bayesian Information Criterion (BIC) is presented for the assessment of the switch-like change in the signal characteristics occurring just before the awakening. Hopfengärtner et al. (2007) design an efficient, robust and fast method based on power spectral analysis techniques for the off-line detection of epileptic seizures in long-term scalp EEG recordings. These methods do not need a supervised learning or training phase, but their performances largely rely on retrospective analysis of the whole data. That is unsuitable for real-time monitoring applications where the changes are expected to be detected as soon as possible.

In this paper, we concentrate on the problem of automatic change detection in real-time EEG monitoring and propose a novel framework for this task. In this framework, joint time-domain features are firstly computed to extract temporal fluctuations of a given EEG data stream; and then, an auto-regressive (AR) linear model is adopted to model the data and temporal anomalies are subsequently calculated from that model to reflect the possibilities that a change occurs; a non-parametric statistical test based on randomized power martingale (RPM) is last performed for performing change decision making based on the resulting anomaly scores.

The rest of the paper is organized as follows. In section 2, the proposed change detection framework is described in details. Experimental results are shown in section 3. We also test the framework in real clinical applications in section 4. A further discussion of the results follows in section 5. Finally, section 6 concludes the paper.

## 2. Materials and methods

### 2.1. Materials

The tested EEG signals in this paper are taken from the publicly-available Bern-Barcelona EEG database (Andrzejak et al., 2012). These EEG data that have been recorded with a sampling rate of 1,024 Hz are down-sampled to 512 Hz prior to further analysis. They randomly select 3,750 pairs of simultaneously recorded signals from the pool of all signals measured at focal and non-focal EEG channels respectively, and divide the recordings into time windows of 20 s, corresponding to 10,240 samples. The first 50 focal and non-focal types of EEG data are selected (note that we only test *x*-signals in given source file).

**Figure 1:**
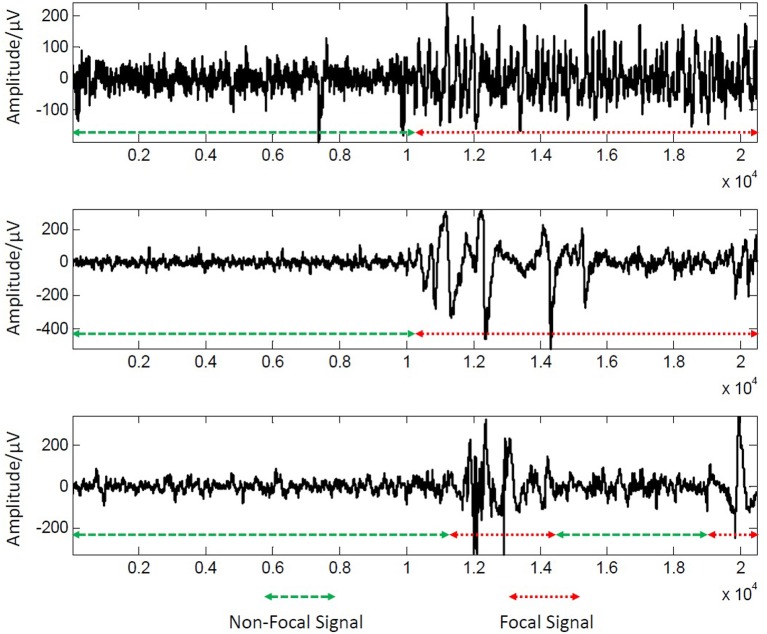
Three examples of testing data streams used in our study.

In our experiment, 50 new EEG data streams were generated by concatenating each pair of a non-focal signal record and a focal signal record to guarantee at least one change point are contained in each testing EEG data stream. We further down-sampled these signals with a down-sampling rate 1:50 in our experiments to decrease the computation burden in process. Finally, two experienced neurologists were invited to label the change point(s) in testing data. Some examples of testing data streams are shown in Figure 1. We conducted the experiment in Matlab R2014a without any optimization for speeding up the procedure, and the PC for the experiment is CPU 3.70 GHz, RAM 4.00 GB.

To evaluate the performance of the proposed change detection framework, we compared the results of automatic detection by our method with those given by experienced neurologists. Three statistical measurements of *precision (specificity), recall (sensitivity)* and *F_score* are used to assess the performance of our approach, which are respectively defined as:

$$\begin{array}{l} \begin{array}{c} \text{precision}=\frac{{\text{n}}_{\text{m}}}{\text{N}} \end{array} \end{array}$$

$$\begin{array}{l} \begin{array}{c} \text{recall}=\frac{{\text{n}}_{\text{m}}}{{\text{N}}_{\text{g}}} \end{array} \end{array}$$

$$\begin{array}{l} \begin{array}{c} \text{F\_score}=\frac{2\times \text{precision}\times \text{recall}}{\text{precision}+\text{recall}} \end{array} \end{array}$$

where *N* is the number of changes from automatic detection by the proposed framework, *N*_*g*_ is the number of all changes labeled by invited neurologists and *n*_*m*_ is the number of changes which are matched-successfully to manual determination from automatic detection. In fact, precision revels the ability to detection accuracy while recall describes the ability to retain or keep accurate and essential information within detected events. Apparently, *F_score* provides a harmonic mean between precision and recall, and a high value of *F_score* ensures reasonably a good balance between them.

### 2.2. Method

In this section, the proposed method will be described in details.

#### 2.2.1. Overview

**Figure 2:**
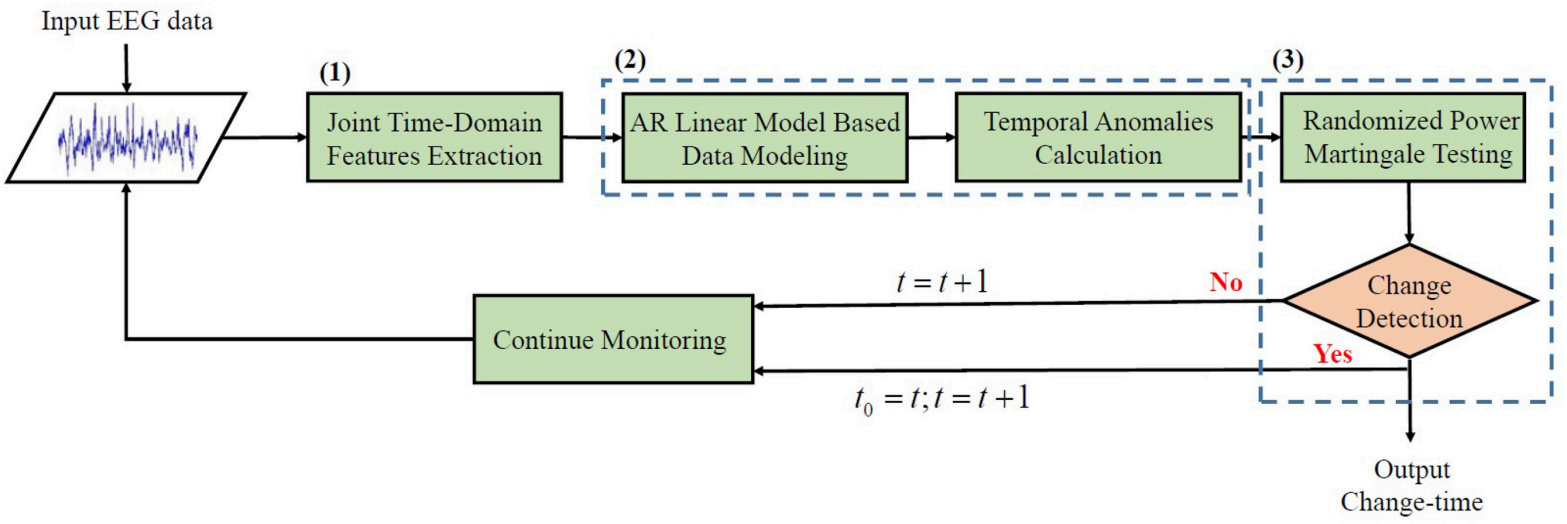
Flowchart of the proposed framework.

As depicted in Figure 2, the framework is operated as follows: (1) joint time-domain features are firstly computed to extract temporal fluctuations of a given EEG data stream; and then, (2) an auto-regressive (AR) linear model is adopted to model the data and temporal anomalies are subsequently calculated from that model to reflect the possibilities that a change occurs; (3) a non-parametric statistical test based on randomized power martingale (RPM) is last performed for performing change decision making based on the resulting anomaly scores. Detailed description will be given in the following.

#### 2.2.2. Feature extraction

Feature extraction for EEG signals representation plays an important role in change detection of the data (Guerrero-Mosquera et al., 2010; Şen and Peker, 2013). In our study, joint time-domain features are used to represent the given EEG signals considering that single time-domain feature may be not reliable enough for the signal representation because of its non-stationary characteristic (Boashash et al., 2015).

For a given EEG data stream $Y=\left\{y_{1},y_{2},\ldots ,y_{N}\right\}$, where *y*_*i*_ is the amplitude of the signal at time *i* and *N* is the length of the stream, we employ a sliding window with a fixed length *L* to extract features (The length of sliding window *L* can be set empirically or with a prior estimation. Here, in this paper, the value of *L* was set as 5 empirically in the following experiments) as follows. For the signal at time *k* (*k* ≥ *L*), we employ five time-domain features *f*_*j*_ (*j* = 1, 2, 3, 4, 5) resulted from the signal sequence within the sliding window {*y*_*k*−*L*+1_, …, *y*_*k*−1_, *y*_*k*_} to generate a joint feature corresponding to this signal^1^. And the expressions and descriptions corresponding to employed time-domain features are listed in Table 1.

**Table 1:** Time-domain features employed for joint feature.

**Feature**	**Expression**	**Description**
*f*_1_	$\frac{1}{L}\sum_{i=k-L+1}^{k}y_{i}$	Mean of EEG signal within the sliding window
*f*_2_	max{*y*_*k*−*L*+1_, …, *y*_*k*_}	Maximum of EEG signal within the sliding window
*f*_3_	min{*y*_*k*−*L*+1_, …, *y*_*k*_}	Minimum of EEG signal within the sliding window
*f*_4_	$\frac{1}{L}\sum_{i=k-L+1}^{k}{\left(y_{i}-f_{1}\right)}^{2}$	Variance of EEG signal within the sliding window
*f*_5_	$\sqrt{\frac{1}{L}\sum_{i=k-L+1}^{k}{\left(y_{i}-f_{1}\right)}^{2}}$	Standard deviation of EEG signal within the sliding window

In fact, we emphasize the amplitude characteristics of given EEG signal via above time-domain features. Here, it is worth mentioning that, the direct use of these resulting statistical features would be straightforward and it would be unreliable to some extent due to its sensitiveness to temporal fluctuations. Hence, the entropy is used to combine these statistical measures as,

(1) $$\begin{array}{l} q_{k}=\overset{5}{\underset{j=1}{\sum }}f_{j}\log f_{j} \end{array}$$

**Figure 3:**
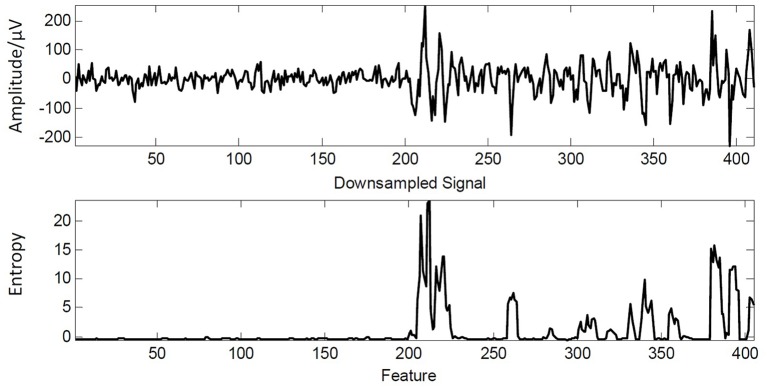
An example of downsampled EEG signal and extracted feature.

Thus, the EEG signal with the length of *N* could be represented as a vector as {*q*_1_, *q*_2_, …, *q*_*N*_}. And an example of extracted feature is given in Figure 3 where the extracted feature can reveal the fluctuation of signal. It shows an obvious representation in where the given signal changes suddenly in amplitude.

Although the resulted feature can point out the time when signal changes largely in time-domain, it is difficult to determine the change based on the calculated entropy because their range can be varied from a small value to a large value. From this perspective, an effective decision rule is still necessary. We will give the detailed detection mechanism in the following.

#### 2.2.3. Problem formulation

We formulate the problem of change detection in this subsection. Suppose that the given EEG signal Y has been represented as {*q*_*t*_}(*t* = 1, 2, …, *N*), we employ an auto-regressive (AR) model to describe the signals without change:

(2) $$\begin{array}{l} q_{t}=\mu +\beta t+\varepsilon_{t} \end{array}$$

where μ and β are the *mean* and *trend* of the EEG series and β is constrained as constant of 0 due to the natural property of EEG signals, that is, the values of EEG data always oscillate around zero, the errors {ε_*t*_} are zero mean, i.e., *E*[ε_*t*_] = 0, which belongs to independent and identical distribution (I.I.D). Hence, we can obtain the expectation of {*q*_*t*_} based on the Equation (2), which is computed as 𝔼[*q*_*t*_] = μ + β*t* + 𝔼[ε_*t*_] = μ + β*t*. In clinical applications, for an EEG sequence $Y=\left\{q_{1},q_{2},\ldots ,q_{N}\right\}$ without obvious change(s), the expectation of {*q*_*t*_} can be estimated approximately as the sample mean as

(3) $$\begin{array}{l} {\bar{q}}_{t}=\frac{1}{N}\overset{N}{\underset{t=1}{\sum }}q_{t}≃\mu +\beta t \end{array}$$

Here, it is worth mentioning that, Equation (3) can be utilized to estimate two important parameters μ and β in Equation (2) by computed ${\bar{q}}_{t}$s.

Assuming that the given EEG signal satisfies the model Equation (2) (i.e., it can be generated from this regression model), we regard that the EEG sequence is a normal state, i.e., no changes occurs, but if it does not, we consider the signal is in an abnormal state, i.e., there is a change in this EEG sequence. Once a change is detected, the current time is determined as one change point. Apparently, the parameters in regression model at change time are different from those resulted from the previous established model. Let us suppose that the model in Equation (2) has been changed from (μ_1_, β_1_) to (μ_2_, β_2_) at time *c*. This change can be described by a two-phase regression model extended from Equation (2) (Wang, 2003), as

(4) $$q_{t}=\{\begin{array}{cc} \mu_{1}+\beta_{1}t+\varepsilon_{t}, & 1\leq t\leq c-1\\ \mu_{2}+\beta_{2}t+\varepsilon_{t}, & c\leq t\leq N \end{array}$$

which allows “*both step(mean)-type* (μ_1_ ≠ μ_2_) and *trend-type* (β_1_ ≠ β_2_)” changes. A null and alternative hypotheses are given as:

(5) $$\begin{array}{l} \begin{array}{ccc} H_{0}:\mu_{1}=\mu_{2} & \text{and} & \beta_{1}=\beta_{2}\\ H_{A}:\mu_{1}\neq \mu_{2} & \text{and/or} & \beta_{1}\neq \beta_{2} \end{array} \end{array}$$

If the null hypothesis *H*_0_ is rejected, i.e., *H*_*A*_ is true, the present time is regarded as a change. Now, the problem is how to quantify the data distribution of Y from this model and discriminate the normal and abnormal states.

#### 2.2.4. Change detection

**Figure 4:**
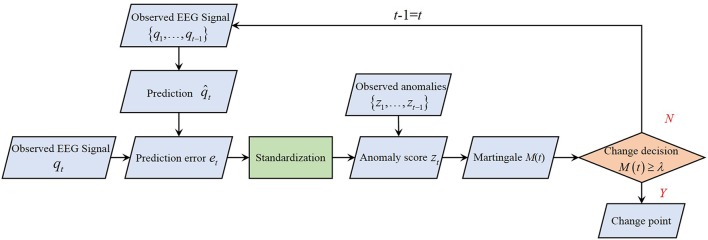
Overview of change detection.

The processing of change detection is shown as Figure 4. A prediction error {*e*_*t*_} is employed to measure the amount of the temporal fluctuations of the EEG data distribution in Y. Assuming that (μ_1_, β_1_) has already been estimated in past signal by Equation (3), the linear prediction ${\hat{q}}_{t}$ at time *t* can be obtained by Equation (2). Denoting the prediction error as *e*_*t*_ at time *t*, we first compute it as

(6) $$\begin{array}{l} e_{t}=||q_{t}-{\hat{q}}_{t}|| \end{array}$$

where || · || is the Euclidean distance metric. Particularly, considering Equations (4) and (6) together, the value of *e*_*t*_ (i.e., *e*_*t*_ = ||Δμ + Δβ*t*|| + ε_*c*_, where Δμ = (μ_1_ − μ_2_) and Δβ = (β_1_ − β_2_)), can be divided into two cases as follows:

- In the case of *t* < *c* where *H*_0_ in Equation (5) is true, *e*_*t*_ is approximately zero or very small, which demonstrates the observed signal obeys the normal data distribution;
- when *t* = *c* which satisfies *H*_*A*_ in Equation (5), *e*_*t*_ will be high which implies a change occurs and the observed data after the change time *c* are considered as another data distribution (i.e., abnormal state).

In order to reduce the computation cost, we use an alternative way to determine $\hat{q}\left(t\right)$ on the basis of Equation (3) as: ${\hat{q}}_{t}\leftarrow {\bar{q}}_{t}$, since the trend β has been constrained as constant of 0 as made previously in this paper. This alternation can effectively speed up the computation of ${\hat{q}}_{t}$ in practical execution because it avoids the large time cost on estimating the parameters μ and β. Moreover, in order to remove the effect of the alternation, we then standardize the resulted {*e*_*t*_} into a series of standardized {*z*_*t*_} by *z*_*t*_ = (*e*_*t*_ − ê)/σ where ê and σ are the sample mean and standard deviation of {*e*_*t*_}. Last, on the basis of {*z*_*t*_}(*t* = 1, 2, …, *N*), we can calculate the anomaly score *s*_*t*_ of each *z*_*t*_ based on the already-observed data {*z*_1_, *z*_2_, …, *z*_*t*−1_} inspired by Ho and Wechsler (2010):

(7) $$\begin{array}{l} s_{t}=s\left(\left\{z_{1},z_{2},\ldots ,z_{t-1}\right\},z_{t}\right)=||z_{t}-H_{t-1}|| \end{array}$$

where $H_{t-1}=\frac{1}{t-1}\overset{t-1}{\underset{i=1}{\sum }}z_{i}$ and || · || is the Euclidean distance metric. On the basis of resulted anomaly scores {*s*_*t*_}(*t* = 1, 2, …, *N*), the current problem is how to find the time *c* when the change occurs.

To achieve automatic detection of the change in {*s*_*t*_}, a non-parametric statistical test based on *randomized power martingale* (RPM) (Vovk et al., 2003) is employed for this task. Detailed computation procedure is described as follows.

First, on the basis of {*s*_1_, *s*_2_, …, *s*_*t*_}, the RPM is constructed by

(8) $$\begin{array}{l} M\left(t\right)=\overset{t}{\underset{i=1}{\prod }}\left(\xi {\hat{p}}_{i}^{\xi -1}\right) \end{array}$$

where ξ ∈ (0, 1) (in the following experiment, it was set as 0.8 since any value of ξ ∈ [0.8, 1) has been investigated the effectiveness in Ho and Wechsler, 2010), and ${\hat{p}}_{i}$s are the ${\hat{p}}_{i}$-values computed from the following function:

(9) $$\begin{array}{l} {\hat{p}}_{i}=\frac{\#\left\{j:s_{j}>s_{i}\right\}+\theta_{i}\#\left\{j:s_{j}=s_{i}\right\}}{i} \end{array}$$

where #{·} is a counting function, *j* ∈ {1, 2, …, *i*−1} and θ_*i*_ is randomly chosen from a uniform distribution of [0, 1] at time *i* (Vovk et al., 2005). It is worth mentioning that, when the data stream includes a change, i.e., the stream does not satisfy the exchangeability, the ${\hat{p}}_{i}$-values using Equation (9) are no longer uniformly distributed in [0, 1] due to the fact that the new data is likely to have higher anomaly scores compared to the data already observed. Here, one observes that, since $M\left(t\right)=\prod_{i=1}^{t}\left(\xi {\hat{p}}_{i}^{\xi -1}\right)$, no re-computation is required for calculating *M*(*t*).

Then, since obviously $\int_{0}^{1}\xi {\hat{p}}_{t}^{\xi -1}={\hat{p}}_{t}^{\xi }{|}_{0}^{1}=1$, the conditional expectation of *M*(*t*), *t* ∈ {1, 2, …, *c*} with respect to the past ${\hat{p}}_{i}$-values, i.e., $\left\{{\hat{p}}_{1},{\hat{p}}_{2},\ldots ,{\hat{p}}_{t}\right\}$ is given by

(10) $$\begin{array}{l} 𝔼\left[M\left(t\right)|{\hat{p}}_{1},{\hat{p}}_{2},\ldots ,{\hat{p}}_{t}\right]=M\left(t-1\right)\int_{0}^{1}\xi {\hat{p}}_{t}^{\xi -1}=M\left(t-1\right) \end{array}$$

This property of “the expectation of the next value is the same as the current value” is so-called *martingale*, which implies *E*[*M*(*t*)] = *E*[*M*(1)] = 1^2^. Suppose that *M*(*t*), *t* ∈ {1, 2, …, *c*} is a nonnegative martingale, the *Doob's Maximal Inequality* (Doob, 1962) is then satisfied for any *t* ∈ {1, 2, …, *c*}:

(11) $$\begin{array}{l} P\left(\underset{0\leq t\leq c}{\max}M\left(t\right)\geq \lambda \right)\leq \frac{1}{\lambda } \end{array}$$

where λ is a positive number. Above inequality shows that not all *M*(*t*)s are higher than a pre-defined threshold, which determines an upper bound for the false alarm rate (i.e., a given probability) for detecting a change when there is none. In other words, the value of λ is determined by the false alarm rate that one is willing to accept (Ho and Wechsler, 2010). However, the false alarm rates in different applications are often chosen by cross-validation or empirical setting. For the decision of martingale test, the Equation (11) can be transformed to the following inequality:

(12) $$\begin{array}{l} 0<M\left(t\right)<\lambda \end{array}$$

The inequality shown in Equation (12) means that, in the martingale-test based change detection, one can reject the null hypothesis *H*_0_ in Equation (5) when *M*(*t*) ≥ λ. In other words, *H*_*A*_: a change occurs on the time *c* as long as *M*(*c*) ≥ λ. This means, decision on a change-point at time *c* is equivalent to testing the following hypothesis by combing Equations (5) and (12) as

(13) $$\begin{array}{l} \begin{array}{l} H_{0}:\;1<M\left(c\right)<\lambda \\ \text{i.e.},\;\mu_{v}^{1}=\mu_{v}^{2}\text{ and }\beta^{1}=\beta^{2}:\text{ no change}\\ H_{A}:\;M\left(c\right)\geq \lambda \\ \text{i.e., }\mu_{v}^{1}\neq \mu_{v}^{2}\text{ and/or }\beta^{1}\neq \beta^{2}:\text{ change occurs} \end{array} \end{array}$$

Once the change is detected, the system will take appropriate actions to maintain the operation and to avoid accidental consequences. Otherwise, the martingale test continues to operate as long as 0 < *M*(*t*) < λ.

#### 2.2.5. Algorithm

Suppose that we have obtained measured anomalies {*s*_*t*_}(*t* = 1, 2, …, *n*), the computation procedure of change detection is given in Algorithm 1 as follows.

**Algorithm 1:** Procedures of change detection.

**STEP 1** Set the value of λ in Eq. (13) for change decision making and initialize the set of changes C=∅; **STEP 2** Set beginning time *t*_0_ = 1 for martingale construction and initialize martingale value *M*(*t*_0_) = 1; **STEP 3** Set time *t* = *t*_0_ + 1 for martingale starting; **STEP 4** Calculate the martingale value by Eq. (8); **STEP 5** Determine the present time by Eq. (13): if it rejects the null hypothesis *H*_0_ via the threshold λ in Eq. (13), add the corresponding time to C and set *M*(*t*) = 1, *t*_0_ = *t, t* = *t*_0_ + 1 successively. After that, goto STEP 4; otherwise, update *t* = *t* + 1 and then goto STEP 4; **STEP 6** The iterative process stops when the monitoring EEG signals Y has been processed completely or at a stopping time.

## 3. Results

**Figure 5:**
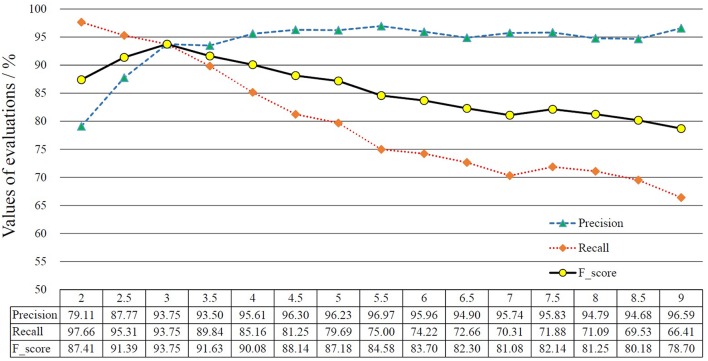
Results of precision, recall and F_score in different values of λ.

**Figure 6:**
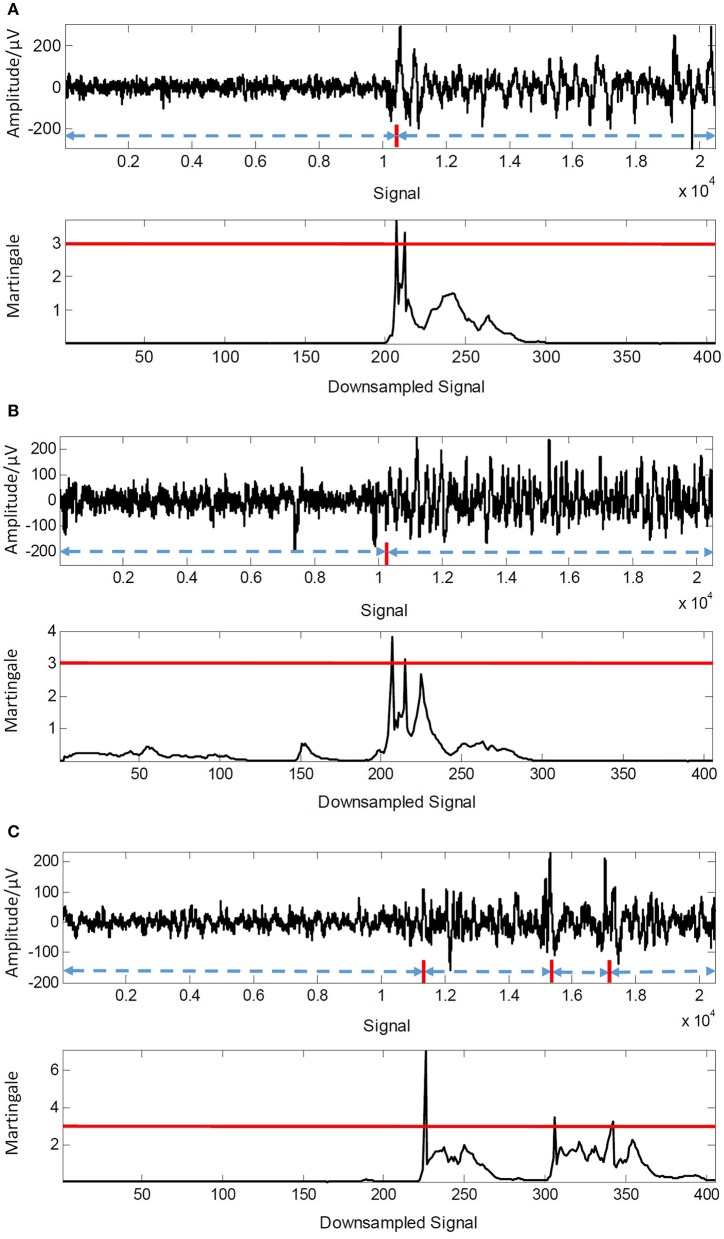
Detection results of three EEG examples when λ = 3. **(A)** Detection result of test signal Ind0005. **(B)** Detection result of test signal Ind0009. **(C)** Detection result of test signal Ind0042.

As the description in Equation (13) that a threshold λ is utilized to control the sensitivity of the detection, we thus gave the results of precision, recall and F_score in different values of λ from 2 to 9 with a step of 0.5 in Figure 5, respectively. The best precision (96.97%) is received when λ = 5.5 while the best recall (97.66%) is obtained when λ = 2. Meanwhile, we can notice that a larger value of precision is performed while a smaller value of recall generates when λ increases. In fact, the value of λ controls the trade-off between precision and recall, and the F_score shows this performance as shown in Figure 5. The F_score receives the most promising value (93.75%) with when λ = 3. We also provided three examples of detection results of different EEG sequences when λ = 3 in Figure 6. The changes labeled manually by neurologists (red line) are shown in top figure in each example. We can notice that only one change is chosen out in the two examples of Figures 6A,B. And three changes in the examples shown in Figure 6C are captured by the framework. The experimental results demonstrate its promising accuracy of detection and ability to keep essential seizure within detected events.

**Figure 7:**
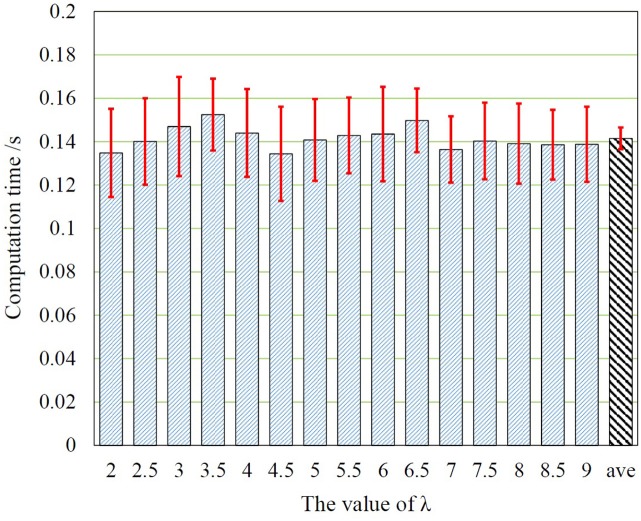
The computation time in different values of λ, where *ave* corresponds to the average computation time of the proposed framework.

For automatic detection in real-time monitoring, the actual processing time is very important for clinical applications. Thus we also presented the computation time in Figure 7. The average computation time is about 0.15*s*. It can be seen that, the phase of change detection is very fast in computation for all tested values of λ, that is faster enough than real-timeness for each EEG signal sequences, which demonstrates the efficiency of our proposed mechanism.

## 4. Application to real EEG monitoring application

**Figure 8:**
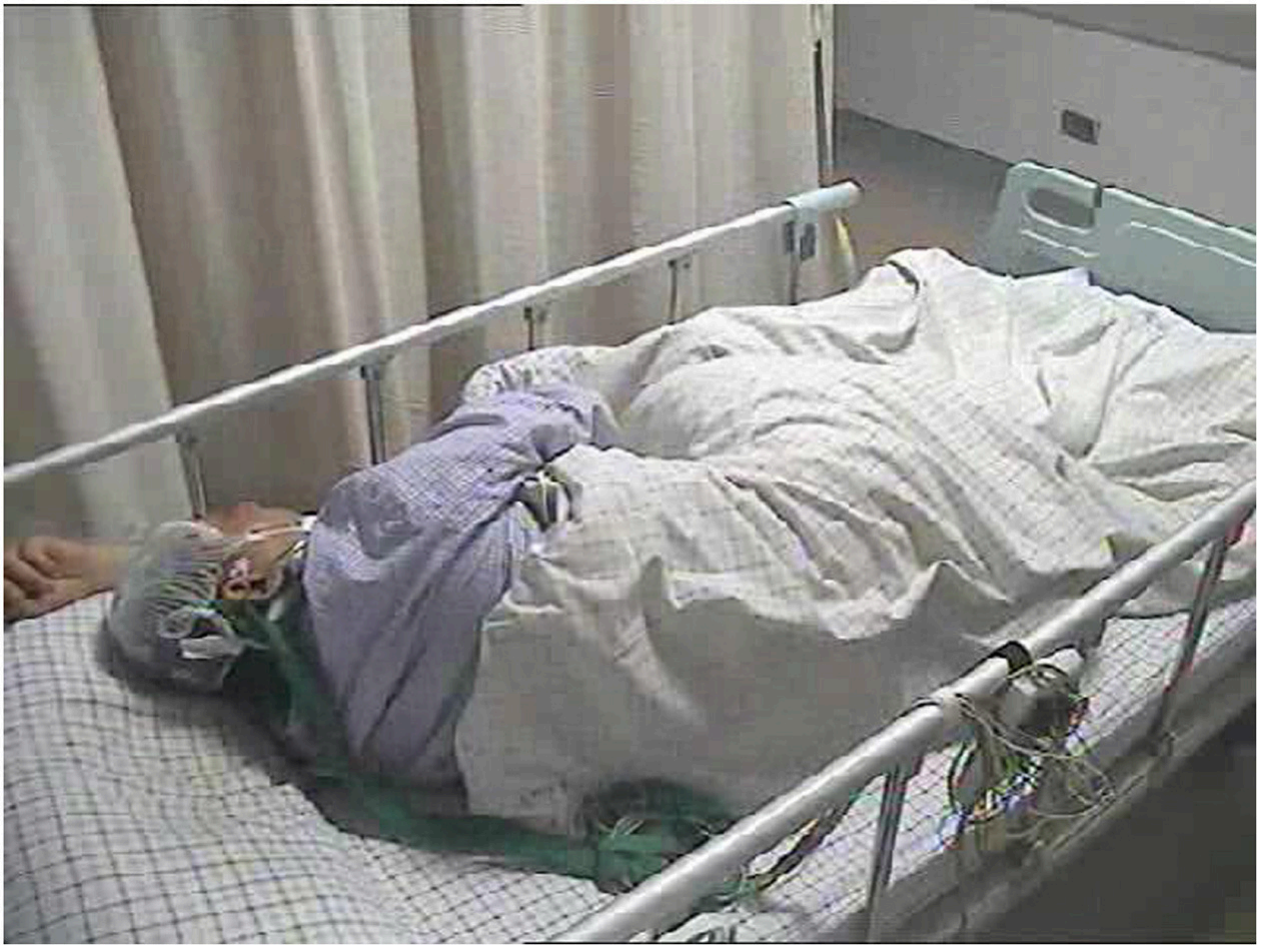
An example of surveillance image in patient monitoring.

**Figure 9:**
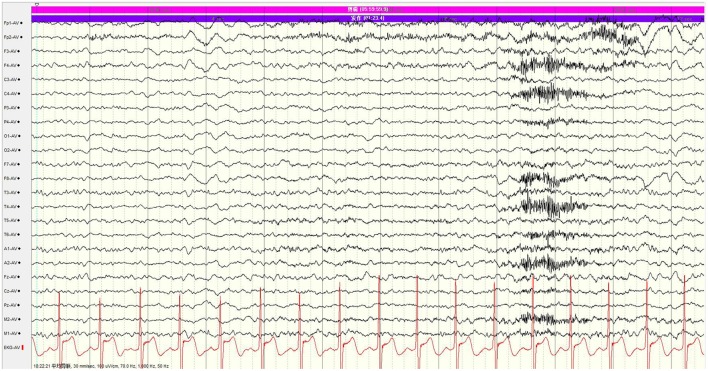
An example of EEG recording when seizure is reported by our framework.

We introduced the proposed framework into the real monitoring of epilepsy seizure based on EEG signals (as shown in Figure 8). The EEG record data and surveillance videos were collected from an epilepsy patient, which lasted for 6 h. The seizure time was recorded by our framework. Meanwhile, an expert observed the EEG recording and labeled the seizure with the assist of monitoring videos. We compared the detection results by our framework with expert's decision. As a result, 30 seizures were detected while there were only 24 seizures given by the expert. We listed the detection results by our framework and expert's decision in Table 2 and showed an example of EEG recording with a seizure reported by our framework in Figure 9. We can notice that six seizures were detected falsely according to expert's decision. On the one hand, the threshold value may be too sensitive for seizure detection (the threshold λ was set as 3 in the experiment). It still needs more clinical testing for determining the suitable threshold. On the other hand, two successive seizure with little time interval was reported by our framework (e.g., 03:22:27 and 03:22:42 in Table 2), which can be regarded as one seizure. As a result, the proposed framework can detect the EEG change effectively although it has a false alarm rate in clinical applications.

**Table 2:** The detection results by the proposed framework:“√” means that the results are endorsed by the expert's decision while “O” means the false detection.

**Seizure**	**Expert**	**Seizure**	**Expert**	**Seizure**	**Expert**	**Seizure**	**Expert**	**Seizure**	**Expert**
00:06:23	√	01:11:07	√	02:42:21	√	03:54:04	√	05:01:32	√
00:19:40	√	01:23:56	√	02:56:03	√	04:06:11	√	05:17:39	√
00:31:12	√	01:33:25	O	03:08:43	√	04:21:29	√	05:29:45	√
00:45:30	√	01:46:10	√	03:22:27	√	04:27:33	O	05:47:25	√
00:56:19	√	02:13:31	√	03:22:42	O	04:48:23	√	05:53:15	O
00:59:23	O	02:29:07	√	03:38:27	√	04:50:52	O	05:54:02	O

## 5. Discussion

Automatic change detection in real-time monitoring of EEG signals is a matter of great significance in theory and clinical practice, which provides an important assistant to observation and diagnosis for the patient undergoing neurological illness. A reliable change detection system can reduce the manual mistakes resulted by neurologists and improve the efficiency.

In this paper, we have proposed a novel framework for automatic change detection in real-time monitoring of EEG signals, which has three key properties:

- *Computational Efficiency*. The framework can be executed very fast, which is experimentally even faster than real-timeness. This property makes it more suitable for change detection in real-time monitoring compared with retrospective analysis based methods (e.g., Saaid et al., 2011; Kortelainen et al., 2012).
- *Unsupervision*. Different from machine learning based approaches (e.g., Kumar et al., 2014; Yuan et al., 2016), our framework does not require any prior knowledge about the EEG signal nor a supervised learning/training phase, which is convenient and simple in real usages.
- *Scalability*. In our framework, change detection can be *from-coarse-to-fine* by only adjusting one parameter λ. Theoretically a smaller number of change will be selected out with a greater value of λ, and vise versa. This achieves a hierarchical analysis/processing of EEG monitoring to meet different clinical demands. For example, a patient with serious brain injuries will need a sensitive detection with a smaller value of λ in order to avoid any omission.

**Figure 10:**
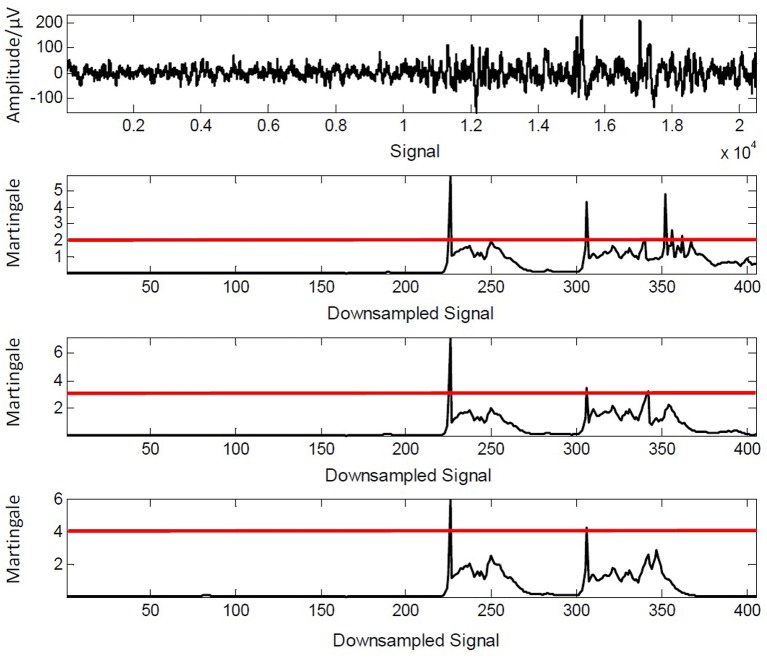
From top to bottom: detection results when λ = 2, 3, 4, respectively.

In detail, it achieves a promising performance of precision (96.97%) and recall (97.66%). Meanwhile, it obtains the best performance of balance between precision (93.75%) and recall (93.75%) when λ = 3. In addition, the computation speed is sufficiently fast to achieve the real-time monitoring and analysis. Note that the framework can be *from-coarse-to-fine* by only adjusting one parameter λ. As seen in an example given in Figure 10, we give the different detection results when λ = 2, 3, 4, respectively. It can be noticed that only one change is captured when λ = 4 while three changes are chosen out when λ = 3. The scalability of our framework provides an adjustable detection for patient with different serious state of neurological illness. For example, the threshold value of λ is supposed to be little for the patient undergoing serious brain injury, because declaring a patient “dead” is a very tricky procedure and it requires a long-time monitoring during which the doctor visually inspects the EEG tracings looking for any change that might account for restart of cerebral activity (La Foresta et al., 2009). They need a more sensitive detection so that any tiny change can not be omitted. In other words, their detection in such a situation needs a larger recall to keep more essential information as much as possible.

Compared with those approaches based on machine learning, our proposed framework is unsupervised. Machine learning based methods such as Cloostermans et al. (2011) and Zhang et al. (2015), often need a supervised learning or training phase, which will be not reliable in the case that we do not have enough prior knowledge or training samples for the learning phase. Moreover, the process of classification/recognition often segments the EEG signals into many epochs with fixed length with an expectation of improving the accuracy of detection, but this strategy is not suitable for real-time applications that require immediate response when the change occurs. On the contrast, our framework determines the state of current time by only using the past data with an online operation way which does not require any prior knowledge about the processing EEG signals so that it can be employed directly for the analysis of new EEG signal with unknown characteristics.

The proposed approach has a high computational efficiency, which brings a much smaller detection delay compared with the results obtained from retrospective analysis (e.g., Saaid et al., 2011; Kortelainen et al., 2012). In some clinical applications where a timely alarm is required, automatic detection with a smaller detection delay is more helpful for medical workers to take actions. Our framework is carried out in an online way, which makes it more suitable for the real-time monitoring and analysis in clinical applications such as the continuous monitoring of coma patient in the intensive care unit (ICU).

## 6. Conclusion

In this paper, we have proposed an efficient, unsupervised and scalable framework for automatic change detection in real-time monitoring of EEG signals. Our main contributions are summarized into two folds: (1) joint time-domain features are used for EEG signals representation, which is able to reveal the fluctuation of signal in amplitude, especially non-stationary EEG signals. (2) real-time change detection is proposed based on RPM, which can be implemented in an online operation without any supervised training phase or prior knowledge. Meanwhile, it has a small detection delay in operation. Through experiments conducted on the Bern-Barcelona EEG database and real clinical application, we demonstrated promising performances of the proposed method indicating that the framework can be effectively applicable in future clinical applications.

## Author contributions

ZG and GL designed the framework and drafted the manuscript. GL, PY, and XL critically revised the content of the work. CL gave much valuable suggestions in the design of framework and helped us revise the manuscript critically. ZG, WS, ZX, and WZ tested the algorithm against the database and real application.

### Conflict of interest statement

The authors declare that the research was conducted in the absence of any commercial or financial relationships that could be construed as a potential conflict of interest.

## Footnotes

^1^Features corresponding to the signal {*y*_1_, *y*_2_, …, *y*_*L*−1_} are initialized as zeros.

^2^*M*(1) is initialized as 1 in settings.
